# Primary Gastrointestinal T-cell Lymphoma Presenting as Spontaneous Perforation Peritonitis

**DOI:** 10.7759/cureus.35028

**Published:** 2023-02-15

**Authors:** James Love, Hasan Raza, Pouyan Kheirkhah, Elie Ghoulam, Sarang Thaker, Robert Carroll

**Affiliations:** 1 Internal Medicine, University of Illinois at Chicago, Chicago, USA; 2 Pathology, University of Illinois at Chicago, Chicago, USA; 3 Gastroenterology and Hepatology, University of Illinois at Chicago, Chicago, USA; 4 Gastroenterology and Hepatology, Northwestern University Feinberg School of Medicine, Chicago, USA; 5 Department of Medicine, University of Illinois at Chicago College of Medicine, Chicago, USA

**Keywords:** t-cell lymphoma, enteroscopy, small bowel, gastrointestinal lymphoma, perforation peritonitis

## Abstract

Primary T-cell non-Hodgkin lymphoma (NHL) of the gastrointestinal tract (GIT) is a rare, poorly-characterized clinical entity. A well-known complication of intestinal NHL is perforation due to chemotherapy, but perforation as a presenting sign of GIT lymphoma is extremely rare. Here we present a case of spontaneous intestinal perforation secondary to primary intestinal T-cell lymphoma and highlight the importance of early recognition of this uncommon cause of perforation as a crucial step to ensure expedited hematology referral and initiation of appropriate treatment.

## Introduction

Primary small bowel neoplasms are incredibly rare, comprising approximately 1-5% of all gastrointestinal cancers in the United States [1-2]. Lymphomas account for only 1-4% of neoplasms in the gastrointestinal tract (GIT), and GIT involvement is usually secondary to widespread nodal disease with approximately 30% occurring within the small intestine. Nearly all primary GI lymphomas are of B-cell origin, with less than 10% incidence of primary T-cell non-Hodgkin lymphoma (NHL) [3]. The initial presenting symptoms of primary T-cell NHL of the GIT are non-specific, with abdominal pain being the most common chief complaint [4]. Perforation is a well-known complication of intestinal NHL due to chemotherapy; however, perforation as the initial presentation of primary GIT lymphoma is extremely rare.

This article was previously presented as a meeting abstract at the 2021 American College of Gastroenterology Annual Meeting on October 25, 2021.

## Case presentation

**Figure 1:**
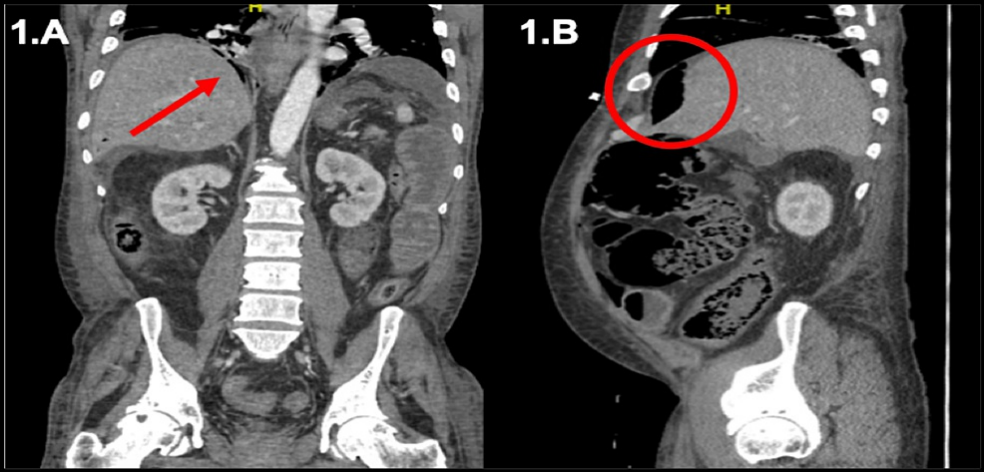
CT Abdomen with thin lucency below the right hemidiaphragm (A) and moderate pneumoperitoneum in the right upper quadrant and anterior abdomen (B) suggestive of moderate pneumoperitoneum. CT = computerized tomography

A 56-year-old man presented with acute severe abdominal pain and nausea. On arrival, he was hypotensive and tachycardic. His abdomen was rigid and diffusely tender. Labs were notable for leukocytosis, lactic acidosis, and venous pH of 7.2. Abdominal computed tomography (CT) demonstrated moderate pneumoperitoneum and high-grade small bowel obstruction without an identifiable transition point, as seen in Figure 1, and he was taken for emergent exploratory laparotomy.

**Figure 2:**
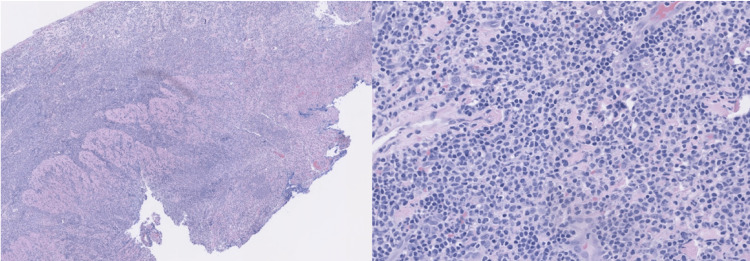
Fragments of tissue from site of perforation with total denudation of the mucosa and gland dropout, ulceration, and marked acute and chronic inflammation.

Intraoperatively, a 3 mm perforation within an inflamed segment of the proximal ileum was found, which was biopsied and sutured closed. Our patient underwent diverting loop ileostomy proximal to the site of inflamed ileum and perforation and was admitted to the intensive care unit (ICU) for postoperative management. Peritoneal culture was positive for E. coli. A biopsy of the perforation showed mucosal denudation, gland dropout, ulceration, and marked acute and chronic inflammation (Figure 2).

**Figure 3:**
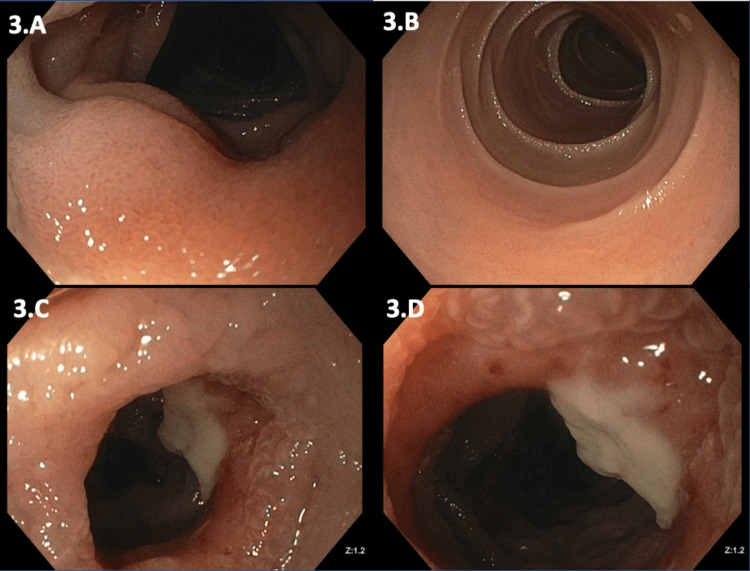
Ileoscopy of ileum notable for scalloped mucosa with blunted villi up to 30 cm proximal to the stoma (A, B) and a single hemi-circumferential 5 mm ulcer without stigmata of recent bleeding (C, D).

**Figure 4:**
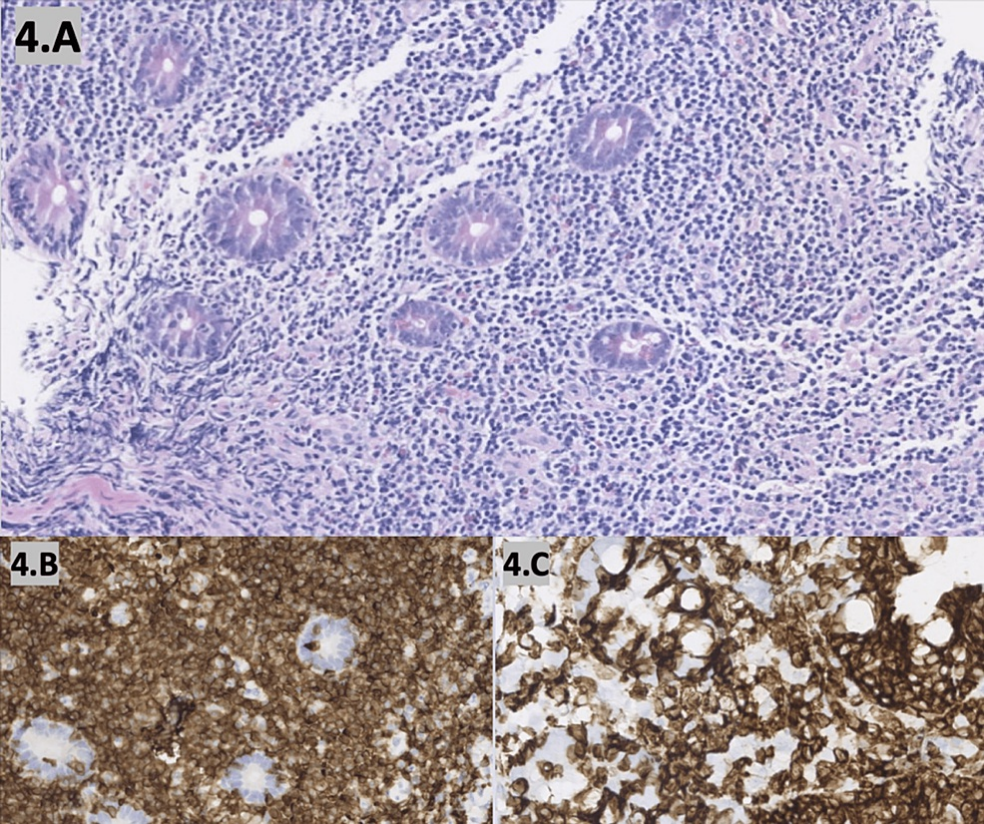
Histopathologic slides of ileal tissue demonstrating focal villous blunting and numerous lymphoid infiltrates composed predominately of small lymphocytes with focal and moderate infiltrates in the lamina propria. Immunohistochemical stains demonstrating that the epitheliotropic lymphocytes are (4B) CD3 and (4C) CD7 positive with variable expression of CD8, confirming T-cell origin.

The postoperative course was complicated by severe protein-calorie malnutrition requiring total parenteral nutrition (TPN) and high ileostomy output. Ileoscopy prior to discharge revealed scalloped mucosa and blunted villi up to 30cm proximal to the stoma and a single hemi-circumferential 5mm ulcer as seen in Figure 3. Biopsies of this area revealed atypical lymphoid infiltrates, suspicious for involvement by mature intestinal T-cell lymphoproliferative neoplasm (Figure 4).

Subsequent T-cell receptor rearrangement studies were positive for a clonal T-cell population, confirming the diagnosis of intestinal T-cell lymphoma. Staging CT was negative for metastatic disease, and bone marrow biopsy and peripheral smear were unremarkable. Our patient was ultimately referred to hematology for initiation of cyclophosphamide, doxorubicin, vincristine, and prednisone (CHOP) chemotherapy. After two cycles of CHOP chemotherapy, our patient had marked improvement in ostomy output; however, our patient was briefly lost to follow-up and ultimately declined to pursue additional treatment of his cancer. After foregoing additional cycles of chemotherapy, his ostomy output increased, and after a brief admission for hypovolemic shock, he was initiated on TPN and discharged to a long-term acute care hospital for the ongoing care of his high-output ostomy.

## Discussion

Although the GIT is the most common site of extranodal involvement of NHL, primary NHL of the GIT is extremely rare, comprising less than 4% of all primary GIT neoplasms [3]. Intestinal primary T-cell NHL represents less than 10% of primary NHL of the GIT and is more aggressive than its B-cell counterpart, with a median five-year survival rate of 23.8% with appropriate treatment compared to 66.3% for low-grade B-cell and 71.3% high-grade B-cell NHL [5]. Further classification of intestinal T-cell lymphoma follows the World Health Organization system, and is divided into four subclasses: enteropathy-associated T-cell lymphoma (EATL), monomorphic epitheliotropic intestinal TCL (MEITL), indolent T-cell lymphoproliferative disorder of the gastrointestinal tract, and intestinal T-cell lymphoma, not otherwise specified (NOS) [6]. EATL is specifically associated with celiac disease and is the most common subclass of primary T-cell NHL, representing approximately two-thirds of reported cases [6-7]. In this case, celiac serologies were negative.

Early recognition of primary intestinal T-cell NHL is challenging, largely due to its rarity and non-specific range of presenting symptoms: fever, abdominal pain, diarrhea, and GI bleeding [8-10]. There is no established role of cross-sectional abdominal imaging in diagnosing primary intestinal NHL, though findings may include mucosal ulceration, bowel wall thickening, or obstruction, which was seen in our patient. On direct visualization via enteroscopy, intestinal lesions may appear as ulcerations or as diffuse mucosal thickening with coarse or fine granular appearance [11]. Unfortunately, these endoscopic findings are present in a variety of inflammatory intestinal diseases and may be mistaken for other more common diagnoses, including Crohn's disease and intestinal tuberculosis [8,12-13]. Thus, definitive diagnosis relies on biopsy and histopathologic analysis. In cases where suspicion for T-cell involvement is high, Roswell Park Memorial Institute (RPMI 1640) medium supplemented with 10% fetal bovine serum is most commonly used to preserve the cytoarchitecture of T-cells and promote cellular incubation and expansion after obtaining tissue biopsies [14]. Fortunately, in our case, the diagnosis could still be made despite biopsies only being preserved in a formalin solution. 

Treatment of primary intestinal NHL is multimodal, most commonly comprising surgery and CHOP chemotherapy. The same treatment approach is applied to both T-cell and B-cell primary intestinal NHL, though patients with T-cell involvement carry a worse prognosis [5]. While it is extremely rare for primary GIT lymphoma to present as spontaneous bowel perforation, it is not an uncommon complication of its treatment with chemotherapy, occurring in approximately 9% of cases with a median time to perforation of approximately 46 days [11,15]. It is unknown whether spontaneous perforation peritonitis as a presenting sign of GIT lymphoma places patients at higher risk for reperforation while receiving chemotherapy. 

## Conclusions

This case report recognizes spontaneous perforation with peritonitis as a potential presenting sign of primary intestinal T-cell NHL. The diagnosis of NHL strictly relies on histopathologic analysis; thus, in patients with spontaneous perforation of unknown etiology, it is crucial to obtain intraoperative biopsies of the perforation and perform an endoscopic evaluation of intestinal mucosa after surgical recovery to evaluate for suspicious lesions. Given the rarity of this subtype of lymphoma, early recognition of this uncommon cause of perforation is essential to ensure expedited hematology referral and initiation of appropriate treatment.

## References

[1] Siegel RL, Miller KD, Fuchs HE, Jemal A (2022). Cancer statistics, 2022. CA Cancer J Clin.

[2] Serour F, Dona G, Birkenfeld S, Balassiano M, Krispin M (1992). Primary neoplasms of the small bowel. J Surg Oncol.

[3] Ghimire P, Wu GY, Zhu L (2011). Primary gastrointestinal lymphoma. World J Gastroenterol.

[4] Li B, Shi YK, He XH (2008). Primary non-Hodgkin lymphomas in the small and large intestine: clinicopathological characteristics and management of 40 patients. Int J Hematol.

[5] Wang GB, Xu GL, Luo GY (2011). Primary intestinal non-Hodgkin's lymphoma: a clinicopathologic analysis of 81 patients. World J Gastroenterol.

[6] Jiang M, Bennani NN, Feldman AL (2017). Lymphoma classification update: T-cell lymphomas, Hodgkin lymphomas, and histiocytic/dendritic cell neoplasms. Expert Rev Hematol.

[7] Zhang JC, Wang Y, Wang XF, Zhang FX (2016). Type I enteropathy-associated T-cell lymphoma in the colon of a 29-year-old patient and a brief literature review. Onco Targets Ther.

[8] Yang H, Zhang H, Liu W (2022). Differential diagnosis of Crohn's disease and ulcerative primary intestinal lymphoma: a scoring model based on a multicenter study. Front Oncol.

[9] Mashayekhi A, Quiroga EF, Margolick JF, Post GR (2022). Intestinal T-cell lymphoma: a rare entity presenting with severe acute upper quadrant pain. Clin Case Rep.

[10] Ara C, Coban S, Kayaalp C, Yilmaz S, Kirimlioglu V (2007). Spontaneous intestinal perforation due to non-Hodgkin's lymphoma: evaluation of eight cases. Dig Dis Sci.

[11] Shirwaikar Thomas A, Schwartz M, Quigley E (2019). Gastrointestinal lymphoma: the new mimic. BMJ Open Gastroenterol.

[12] Wu PH, Chu KE, Lin YM, Huang SH, Wu CC (2015). T-cell lymphomas presenting as colon ulcers and eosinophilia. Case Rep Gastroenterol.

[13] Sun ZH, Zhou HM, Song GX, Zhou ZX, Bai L (2014). Intestinal T-cell lymphomas: a retrospective analysis of 68 cases in China. World J Gastroenterol.

[14] MacPherson S, Keyes S, Kilgour MK (2022). Clinically relevant T cell expansion media activate distinct metabolic programs uncoupled from cellular function. Mol Ther Methods Clin Dev.

[15] Vaidya R, Habermann TM, Donohue JH (2013). Bowel perforation in intestinal lymphoma: incidence and clinical features. Ann Oncol.

